# Supplementary material for: Bayesian Rank-Clustering
Source: Psychometrika. 2025 Jun 16;90(3):904–31. doi: 10.1017/psy.2025.10014 (PMC12483714; doi:10.1017/psy.2025.10014)
Supplement: Pearce and Erosheva supplementary material [file S0033312325100148sup001.zip › Figures/mayor_comp.pdf]

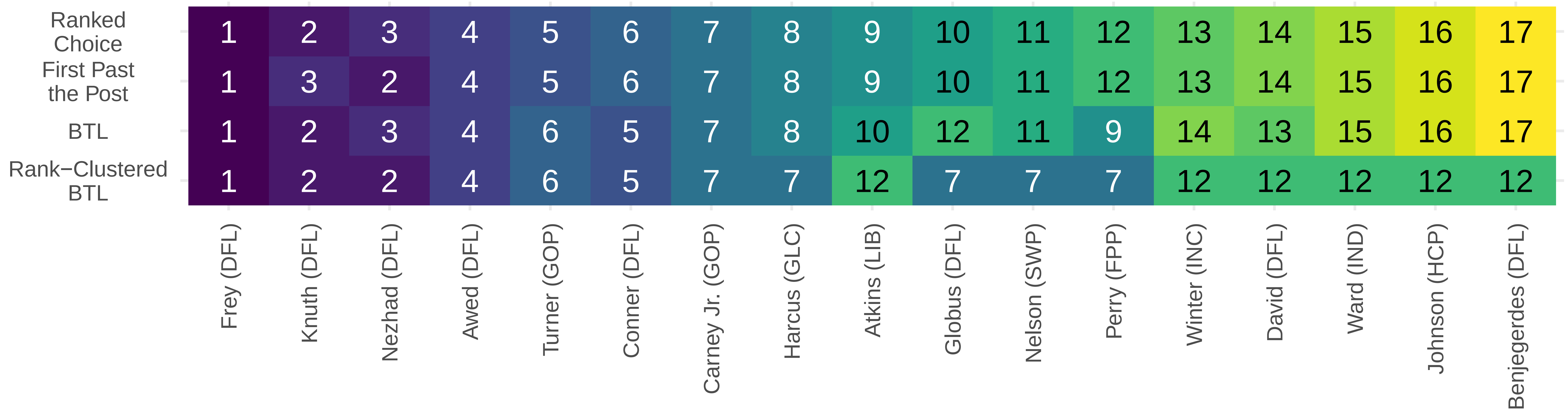
Ranked Choice
1 2 3 4 5 6 7 8 9 10 11 12 13 14 15 16 17
First Past the Post
1 3 2 4 5 6 7 8 9 10 11 12 13 14 15 16 17
BTL
1 2 3 4 6 5 7 8 10 12 11 9 14 13 15 16 17
Rank−Clustered BTL
1 2 2 4 6 5 7 7 12 7 7 7 12 12 12 12 12
Frey (DFL)
Knuth (DFL)
Nezhad (DFL)
Awed (DFL)
Turner (GOP)
Conner (DFL)
Carney Jr. (GOP)
Harcus (GLC)
Atkins (LIB)
Globus (DFL)
Nelson (SWP)
Perry (FPP)
Winter (INC)
David (DFL)
Ward (IND)
Johnson (HCP)
Benjegerdes (DFL)
