# Supplementary material for: Bayesian Rank-Clustering
Source: Psychometrika. 2025 Jun 16;90(3):904–31. doi: 10.1017/psy.2025.10014 (PMC12483714; doi:10.1017/psy.2025.10014)
Supplement: Pearce and Erosheva supplementary material [file S0033312325100148sup001.zip › Figures/NBA_comp.pdf]

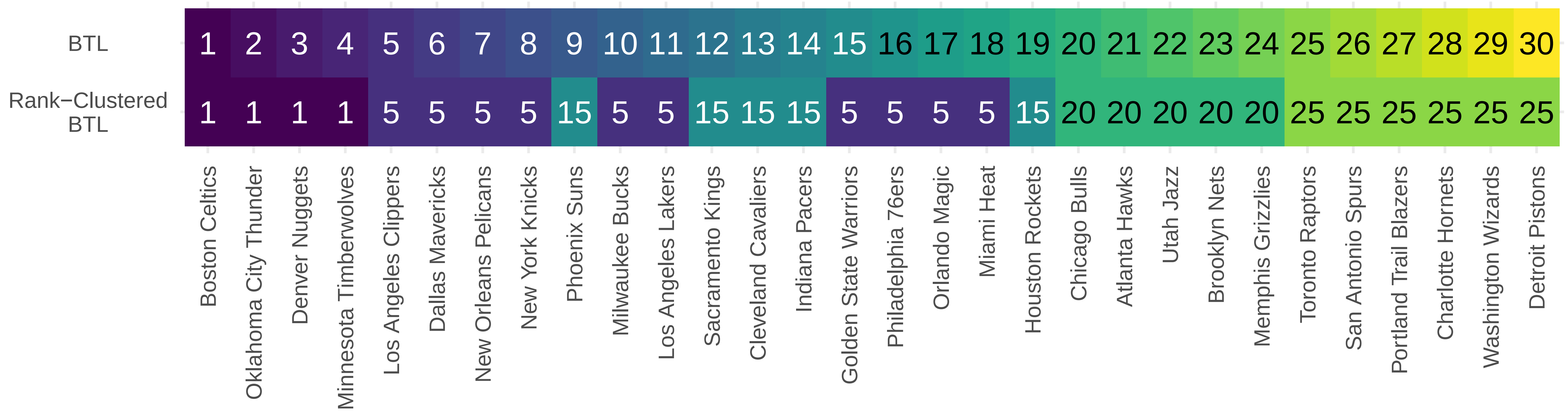

BTL
Rank−Clustered BTL
1 2 3 4 5 6 7 8 9 10 11 12 13 14 15 16 17 18 19 20 21 22 23 24 25 26 27 28 29 30
1 1 1 1 5 5 5 5 15 5 5 15 15 15 5 5 5 5 15 20 20 20 20 20 25 25 25 25 25 25
Boston Celtics
Oklahoma City Thunder
Denver Nuggets
Minnesota Timberwolves
Los Angeles Clippers
Dallas Mavericks
New Orleans Pelicans
New York Knicks
Phoenix Suns
Milwaukee Bucks
Los Angeles Lakers
Sacramento Kings
Cleveland Cavaliers
Indiana Pacers
Golden State Warriors
Philadelphia 76ers
Orlando Magic
Miami Heat
Houston Rockets
Chicago Bulls
Atlanta Hawks
Utah Jazz
Brooklyn Nets
Memphis Grizzlies
Toronto Raptors
San Antonio Spurs
Portland Trail Blazers
Charlotte Hornets
Washington Wizards
Detroit Pistons
