# Supplementary material for: Bayesian Rank-Clustering
Source: Psychometrika. 2025 Jun 16;90(3):904–31. doi: 10.1017/psy.2025.10014 (PMC12483714; doi:10.1017/psy.2025.10014)
Supplement: Pearce and Erosheva supplementary material [file S0033312325100148sup001.zip › Figures/sim_RCProb.pdf]

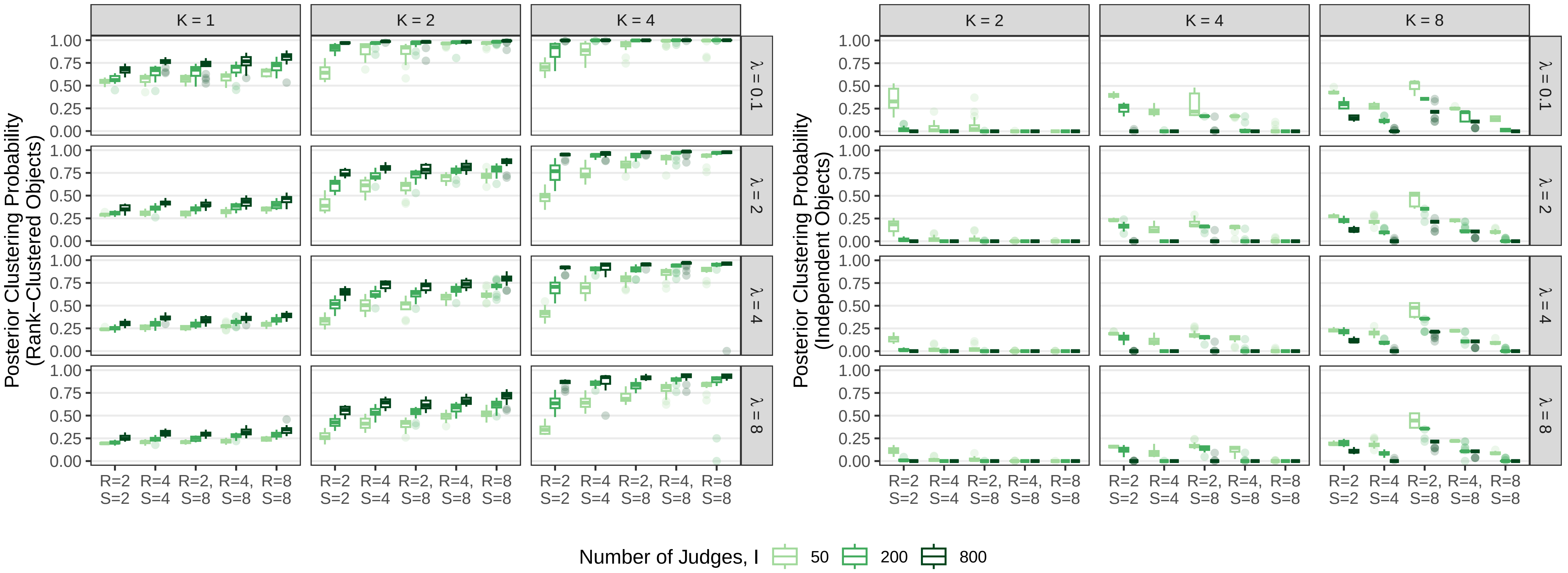
K = 1
K = 2
K = 4
K = 2
K = 4
K = 8
λ = 0.1
λ = 2
λ = 4
λ = 8
Posterior Clustering Probability (Rank−Clustered Objects)
Posterior Clustering Probability (Independent Objects)
1.00
0.75
0.50
0.25
0.00
R=2 S=2
R=4 S=4
R=2, S=8
R=4, S=8
R=8 S=8
Number of Judges, I
50
200
800
