# Supplementary material for: Bayesian Rank-Clustering
Source: Psychometrika. 2025 Jun 16;90(3):904–31. doi: 10.1017/psy.2025.10014 (PMC12483714; doi:10.1017/psy.2025.10014)
Supplement: Pearce and Erosheva supplementary material [file S0033312325100148sup001.zip › Figures/sushi_comp.pdf]

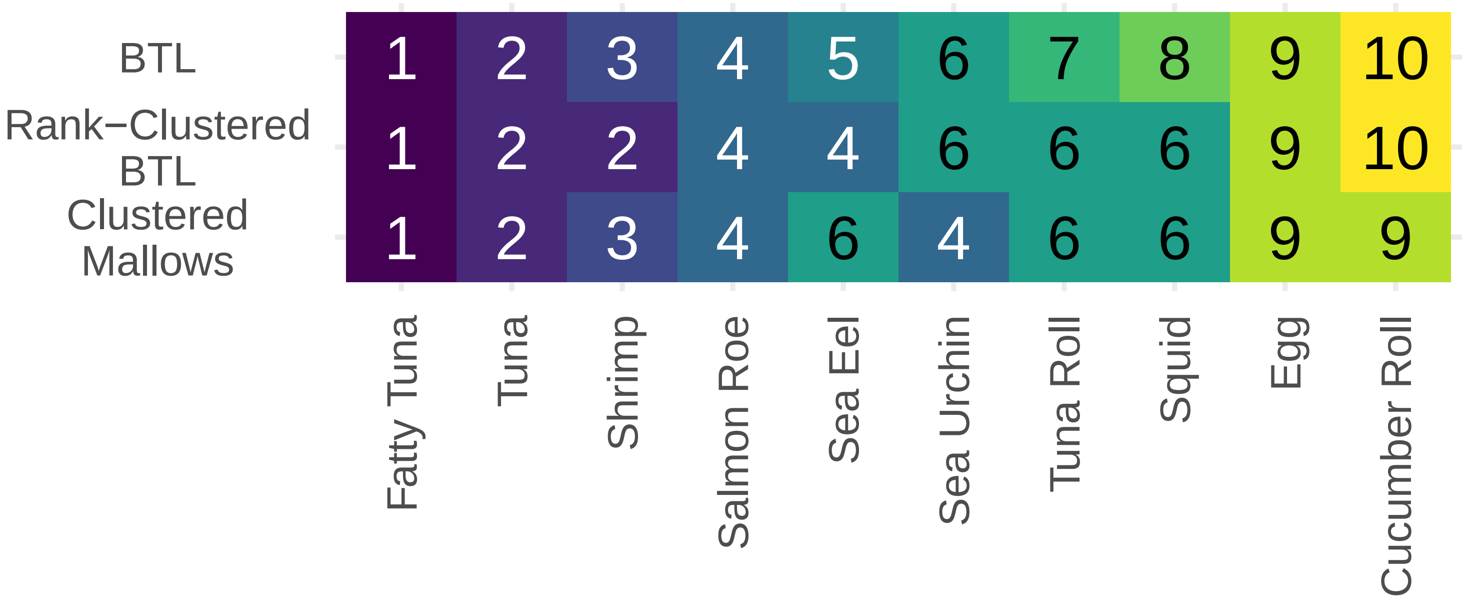
BTL
Rank−Clustered BTL
Clustered Mallows
1 2 3 4 5 6 7 8 9 10
1 2 2 4 4 6 6 6 9 10
1 2 3 4 6 4 6 6 9 9
Fatty Tuna
Tuna
Shrimp
Salmon Roe
Sea Eel
Sea Urchin
Tuna Roll
Squid
Egg
Cucumber Roll
