# Supplementary material for: Bayesian Rank-Clustering
Source: Psychometrika. 2025 Jun 16;90(3):904–31. doi: 10.1017/psy.2025.10014 (PMC12483714; doi:10.1017/psy.2025.10014)
Supplement: Pearce and Erosheva supplementary material [file S0033312325100148sup001.zip › README.rtf]

This folder contains .R code to replicate all analyses presented in “Bayesian Rank-Clustering” by Pearce and Erosheva (2025). The folder contains 7 subfolders:	+ Folders starting with a number contain code to replicate the analyses in a section of the manuscript, e.g., “3.1 PSSF Prior” replicates the analysis of section 3.1. Each folder contains a file named “main.R”, which may be run directly to replicate the analysis and any data required to replicate the analysis.	+ The folder “Figures” is where all figures created in the previous folders are saved.
