# Supplementary figures and images for: Bayesian Rank-Clustering
Source: Psychometrika. 2025 Jun 16;90(3):904–31. doi: 10.1017/psy.2025.10014 (PMC12483714; doi:10.1017/psy.2025.10014)

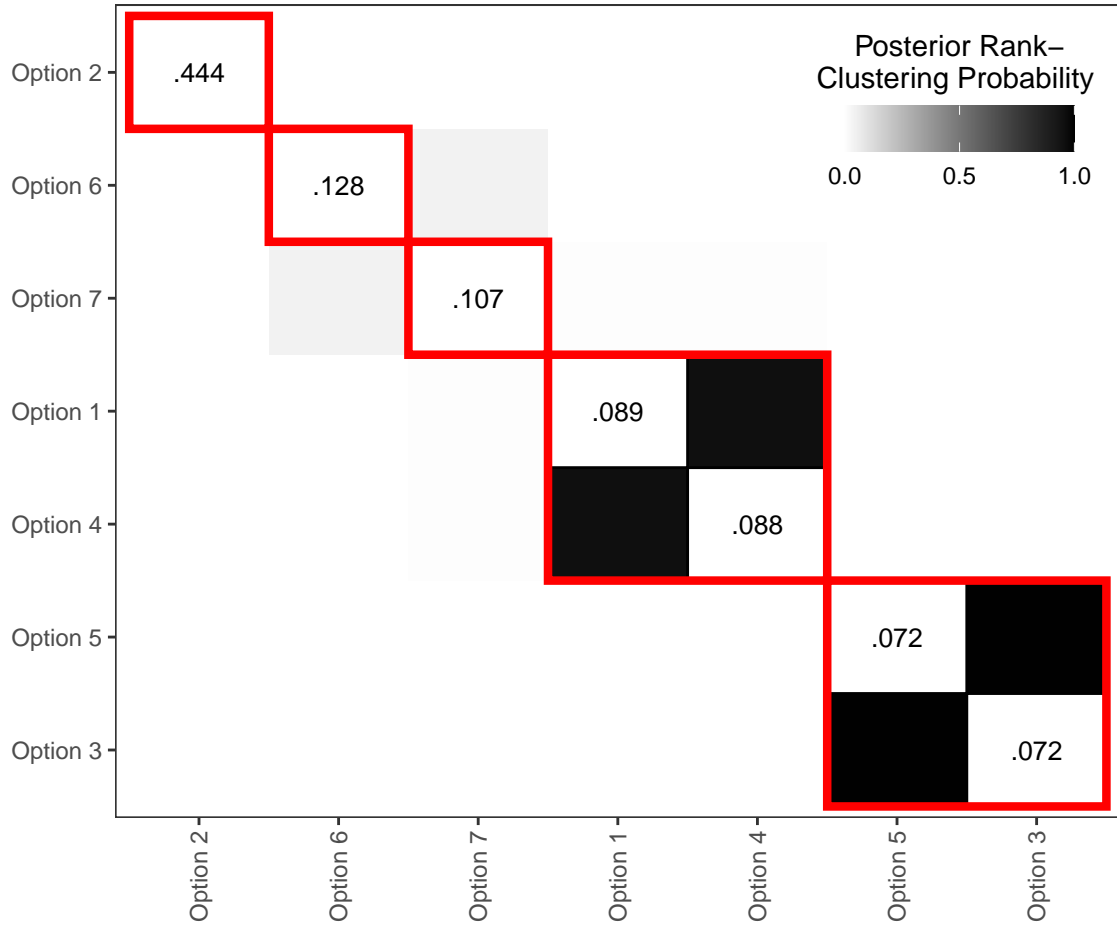

Supplement: Pearce and Erosheva supplementary material [file S0033312325100148sup001.zip › Figures/eurobarometer_cluster.pdf]

BTL

Rank-Clustered  
BTL

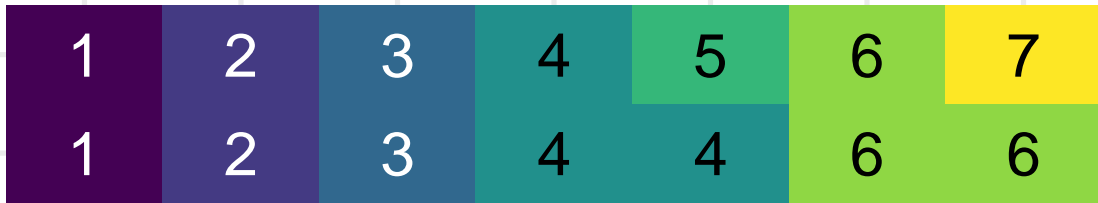

Option 2

Option 6

Option 7

Option 1

Option 4

Option 5

Option 3

Supplement: Pearce and Erosheva supplementary material [file S0033312325100148sup001.zip › Figures/eurobarometer_comp.pdf]

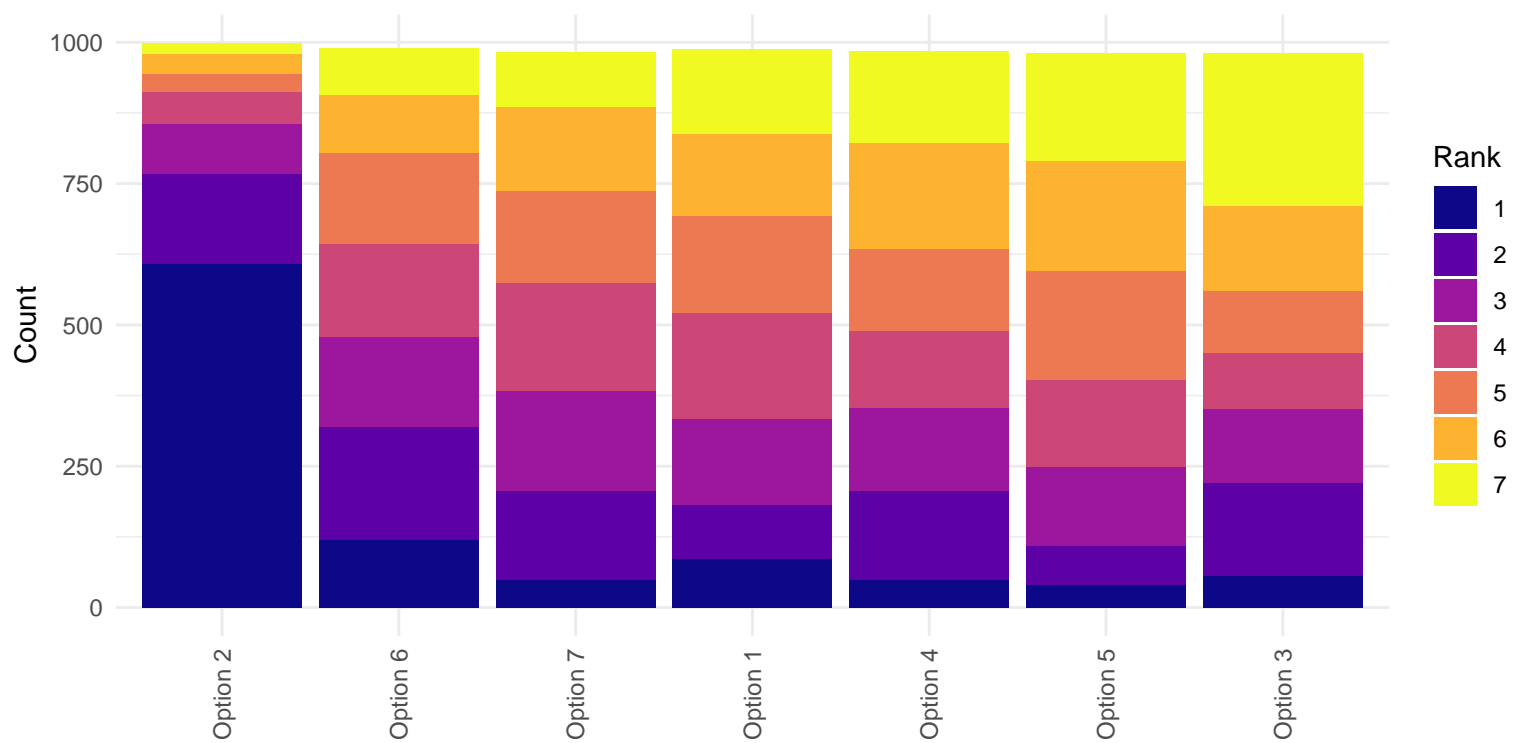

Supplement: Pearce and Erosheva supplementary material [file S0033312325100148sup001.zip › Figures/eurobarometer_EDA.pdf]

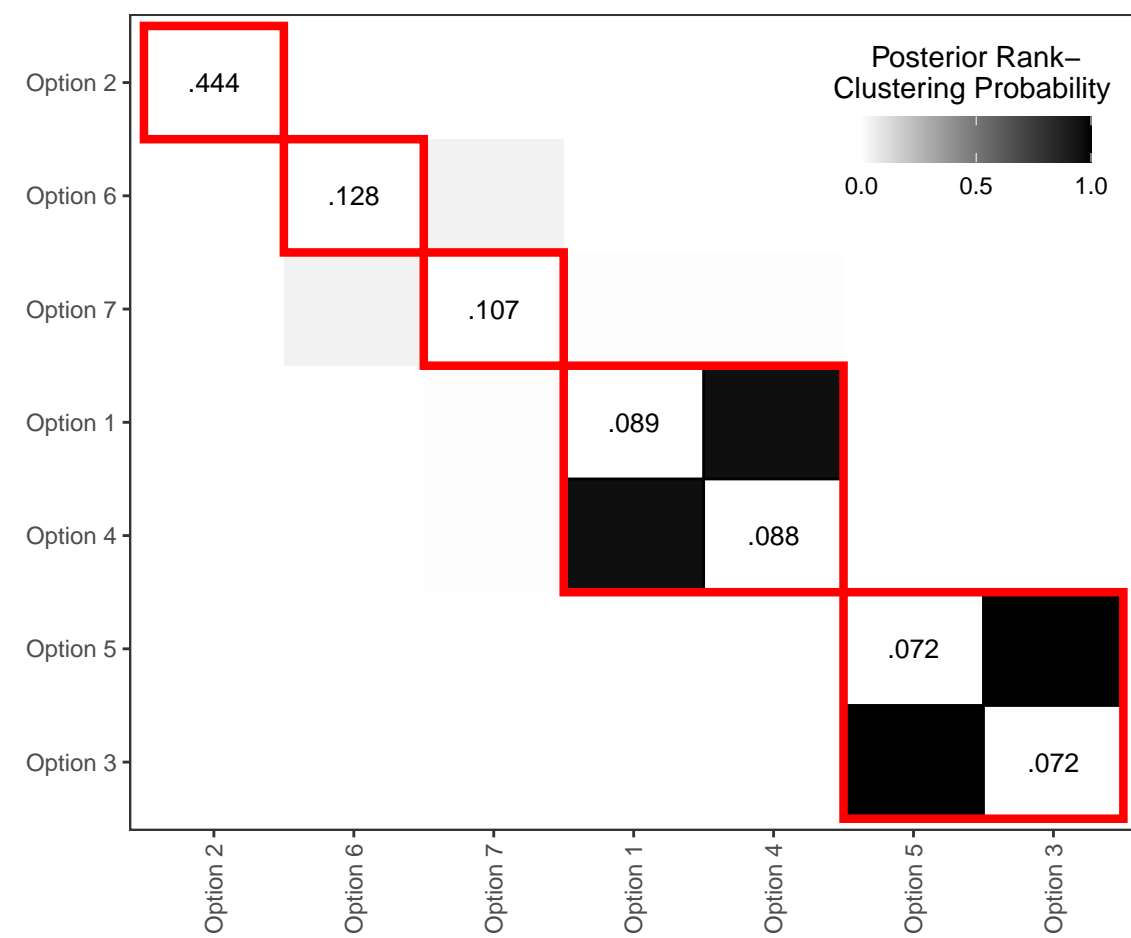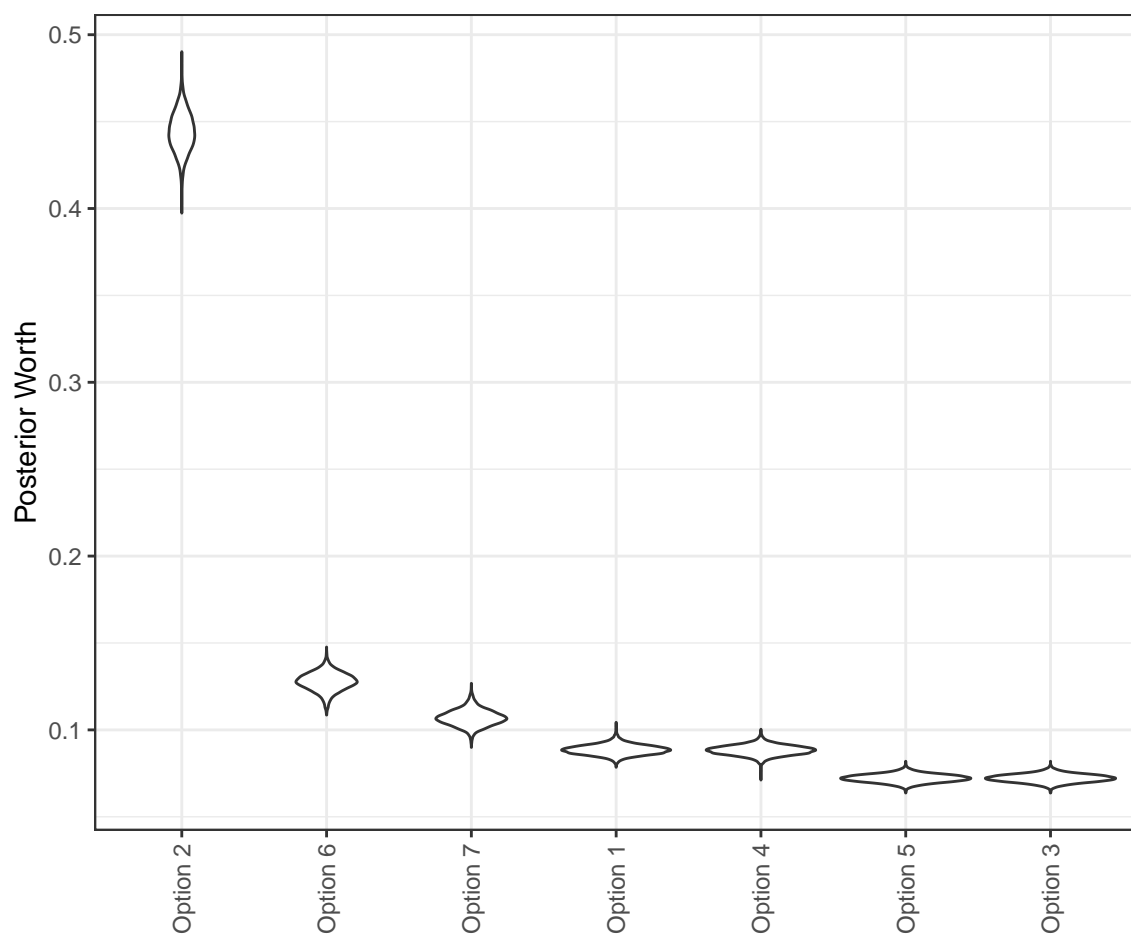

Supplement: Pearce and Erosheva supplementary material [file S0033312325100148sup001.zip › Figures/eurobarometer_main.pdf]

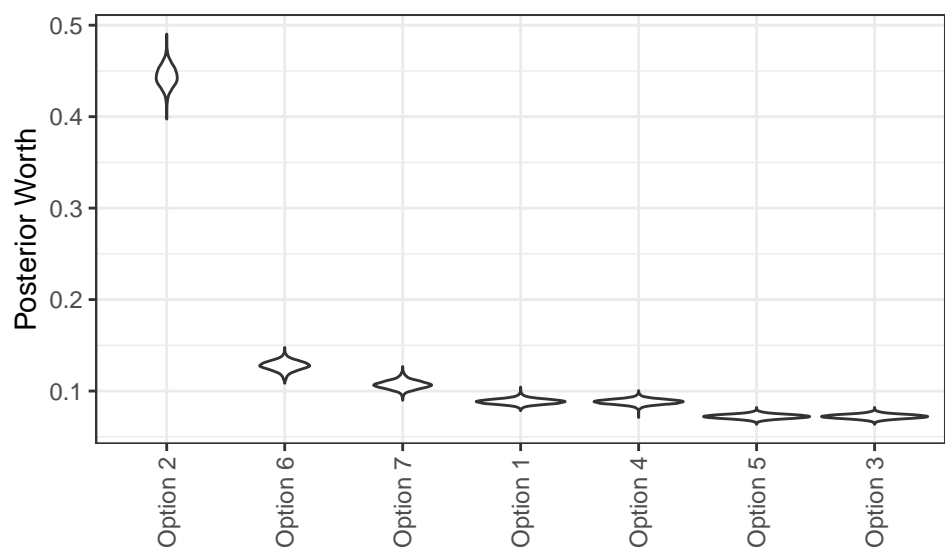

Supplement: Pearce and Erosheva supplementary material [file S0033312325100148sup001.zip › Figures/eurobarometer_posterior.pdf]

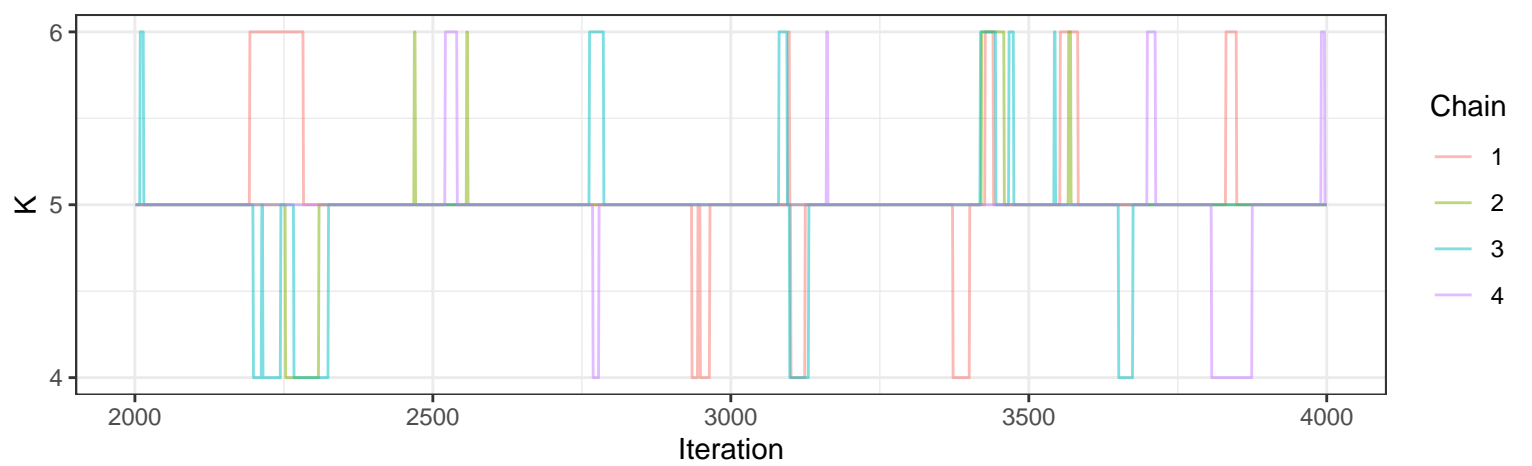

Supplement: Pearce and Erosheva supplementary material [file S0033312325100148sup001.zip › Figures/eurobarometer_traceK.pdf]

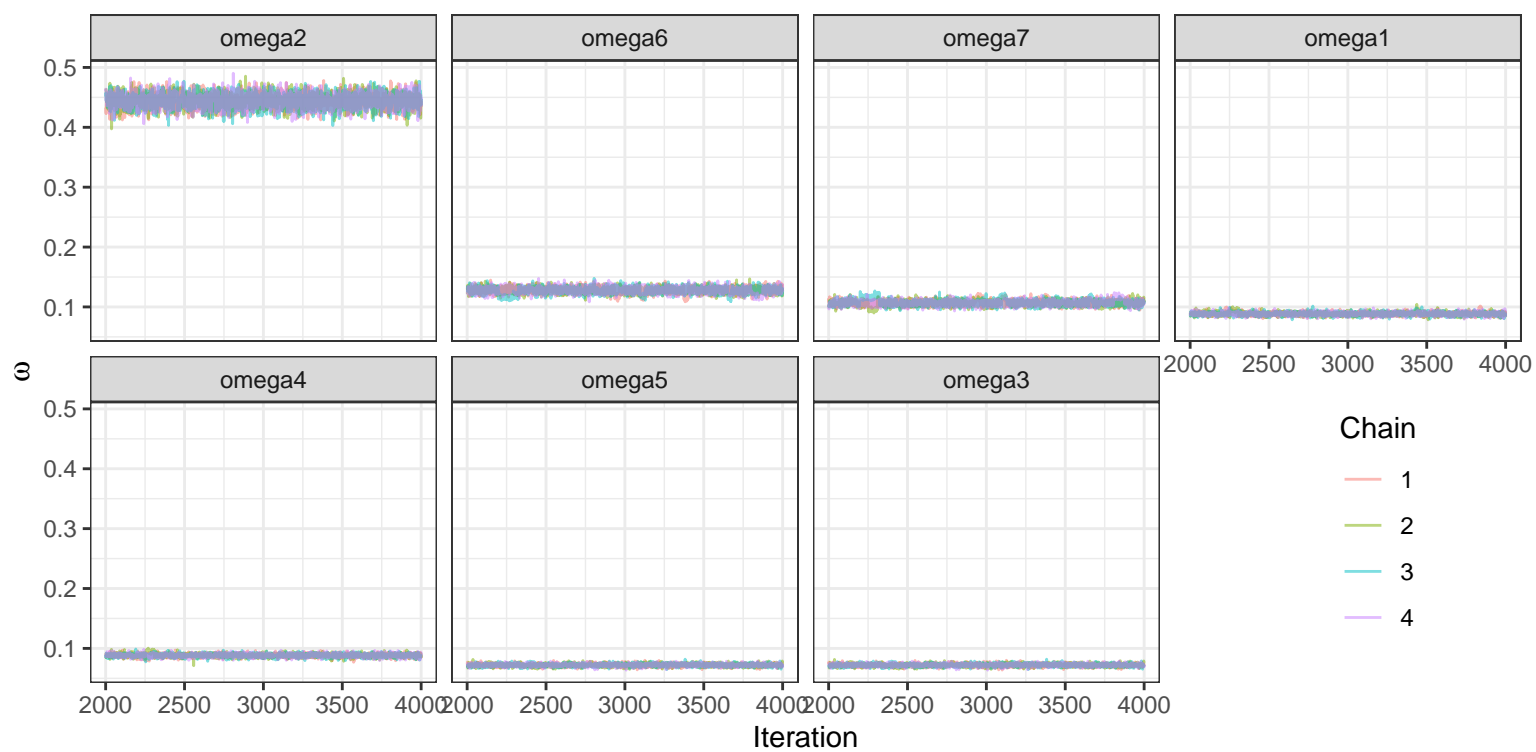

Supplement: Pearce and Erosheva supplementary material [file S0033312325100148sup001.zip › Figures/eurobarometer_traceOmega.pdf]

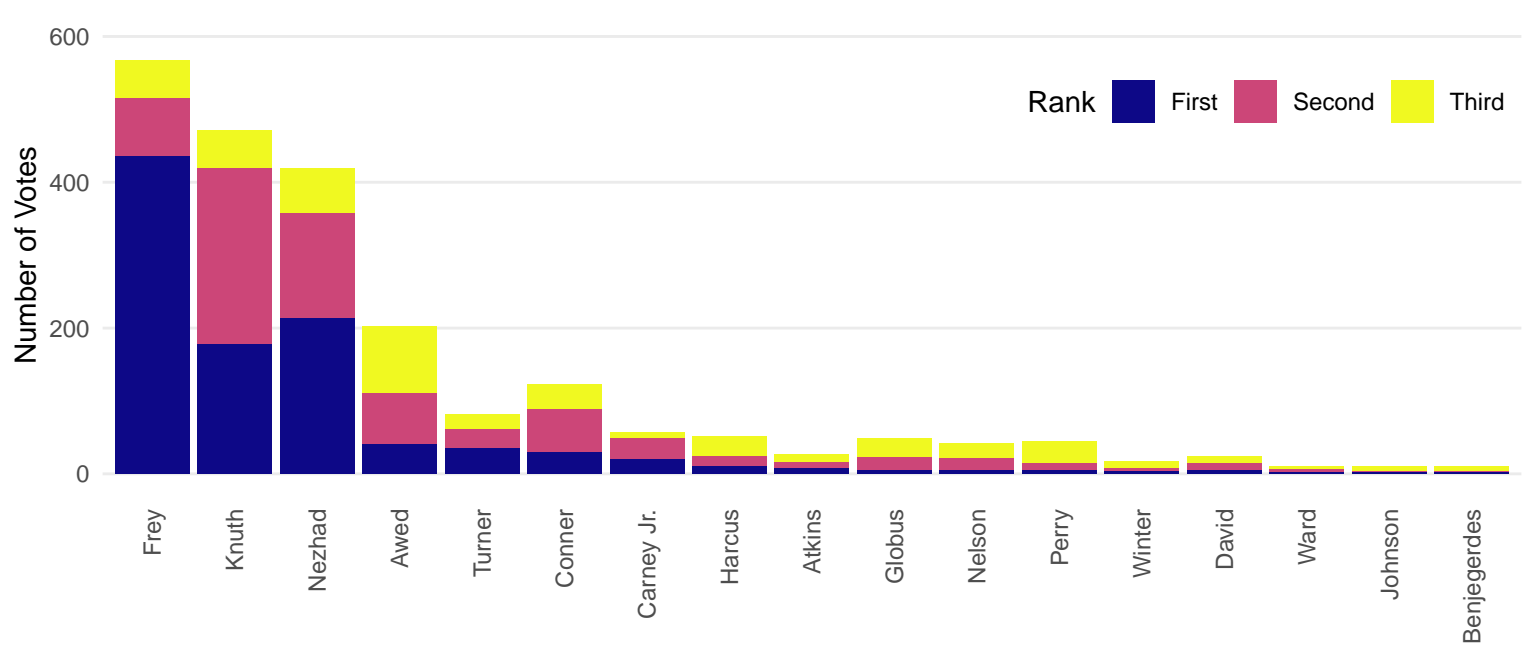

Supplement: Pearce and Erosheva supplementary material [file S0033312325100148sup001.zip › Figures/mayor_EDA.pdf]

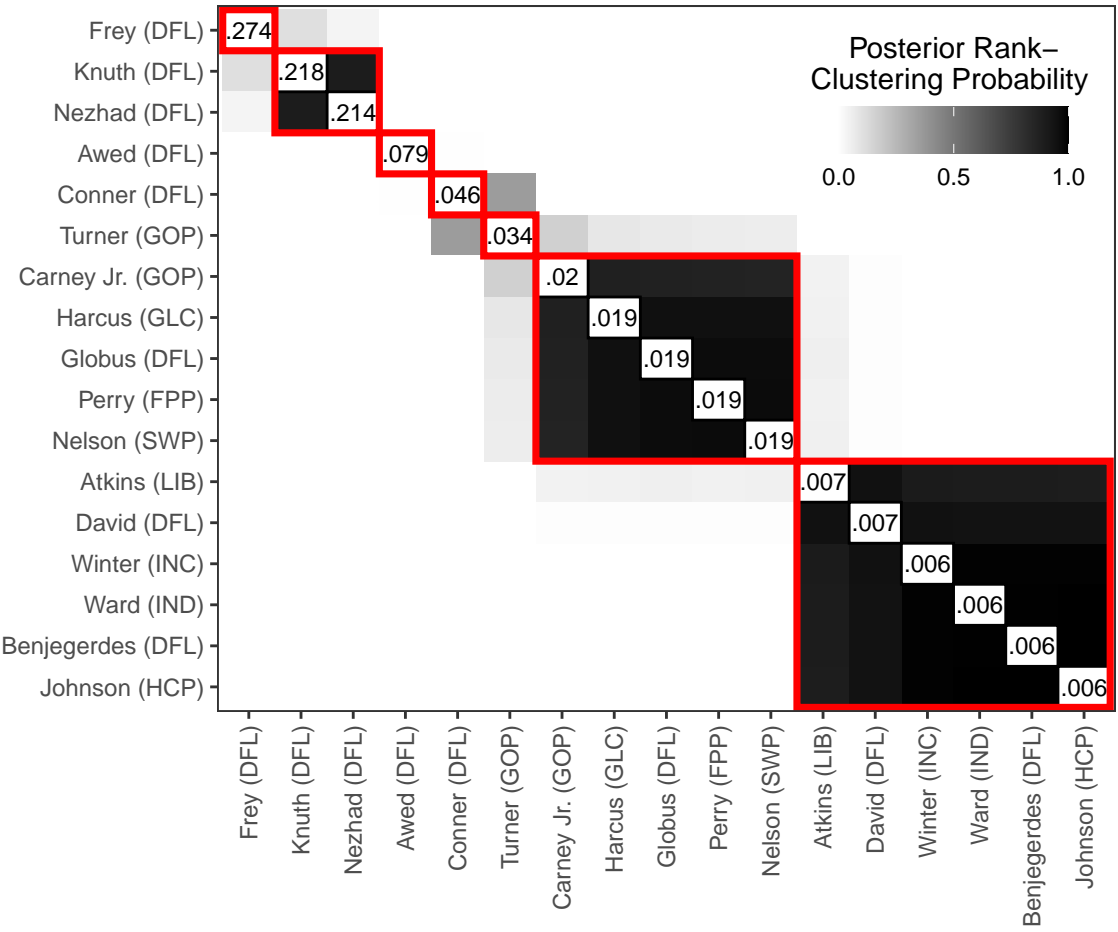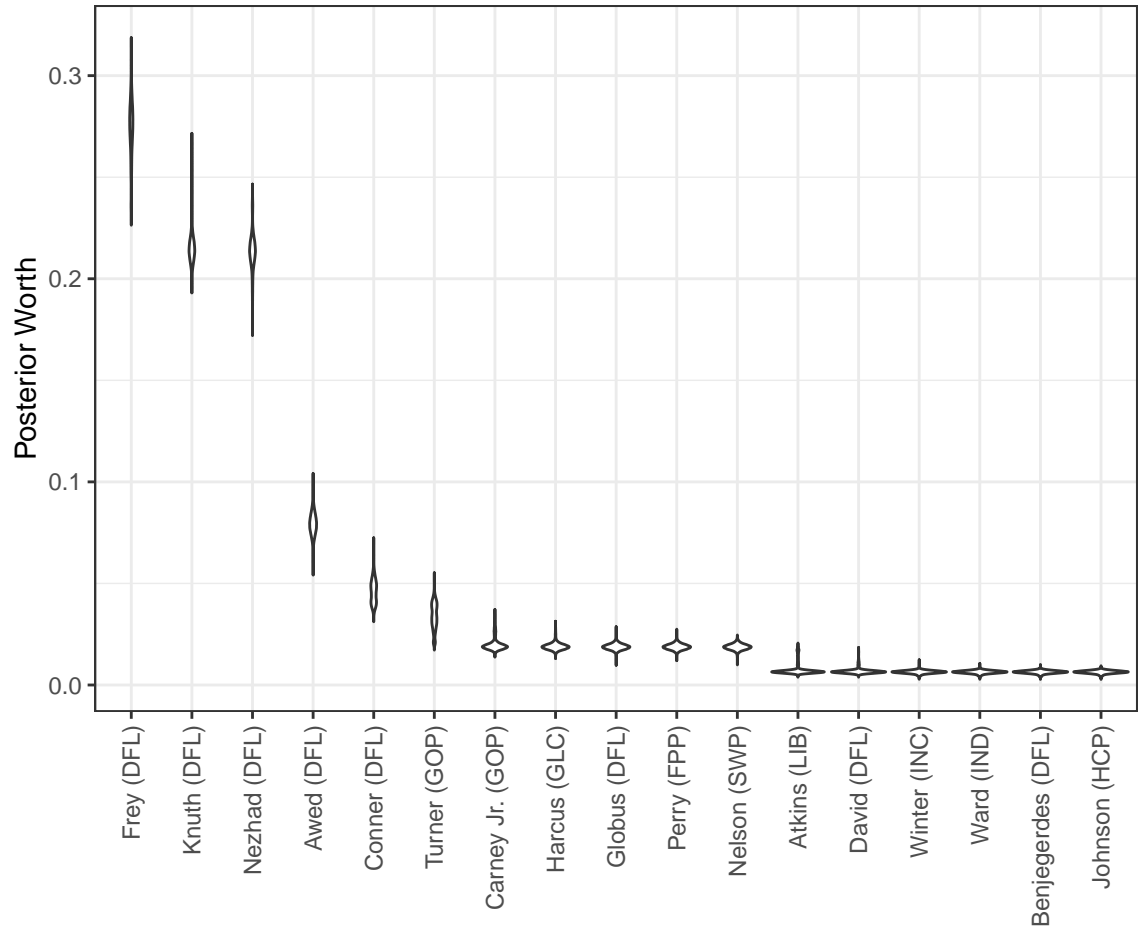

Supplement: Pearce and Erosheva supplementary material [file S0033312325100148sup001.zip › Figures/mayor_main.pdf]

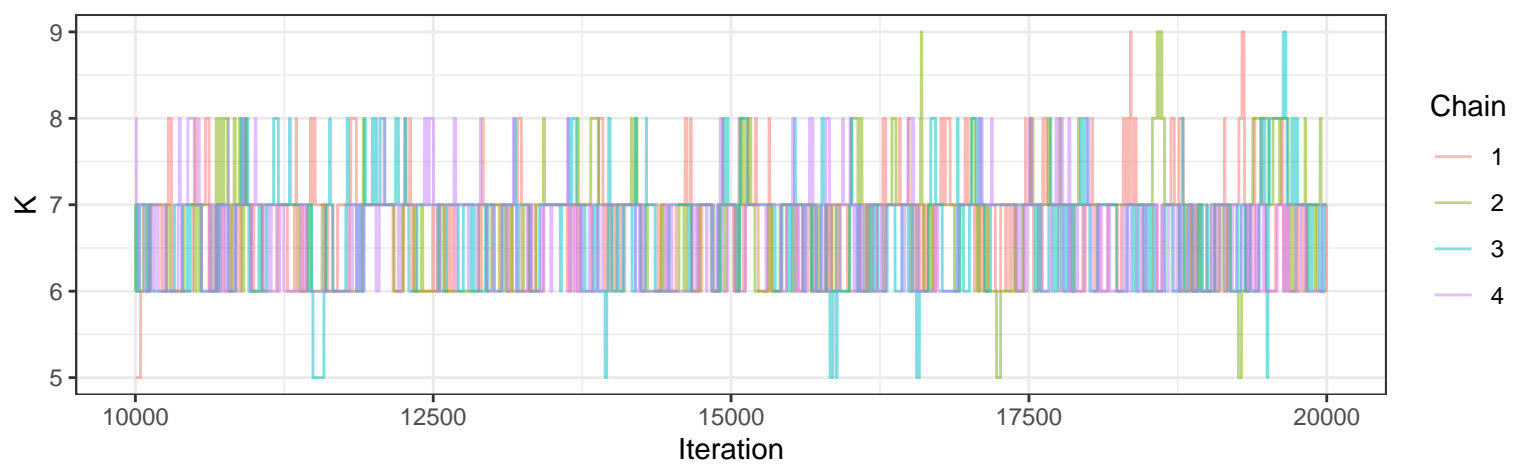

Supplement: Pearce and Erosheva supplementary material [file S0033312325100148sup001.zip › Figures/mayor_traceK.pdf]

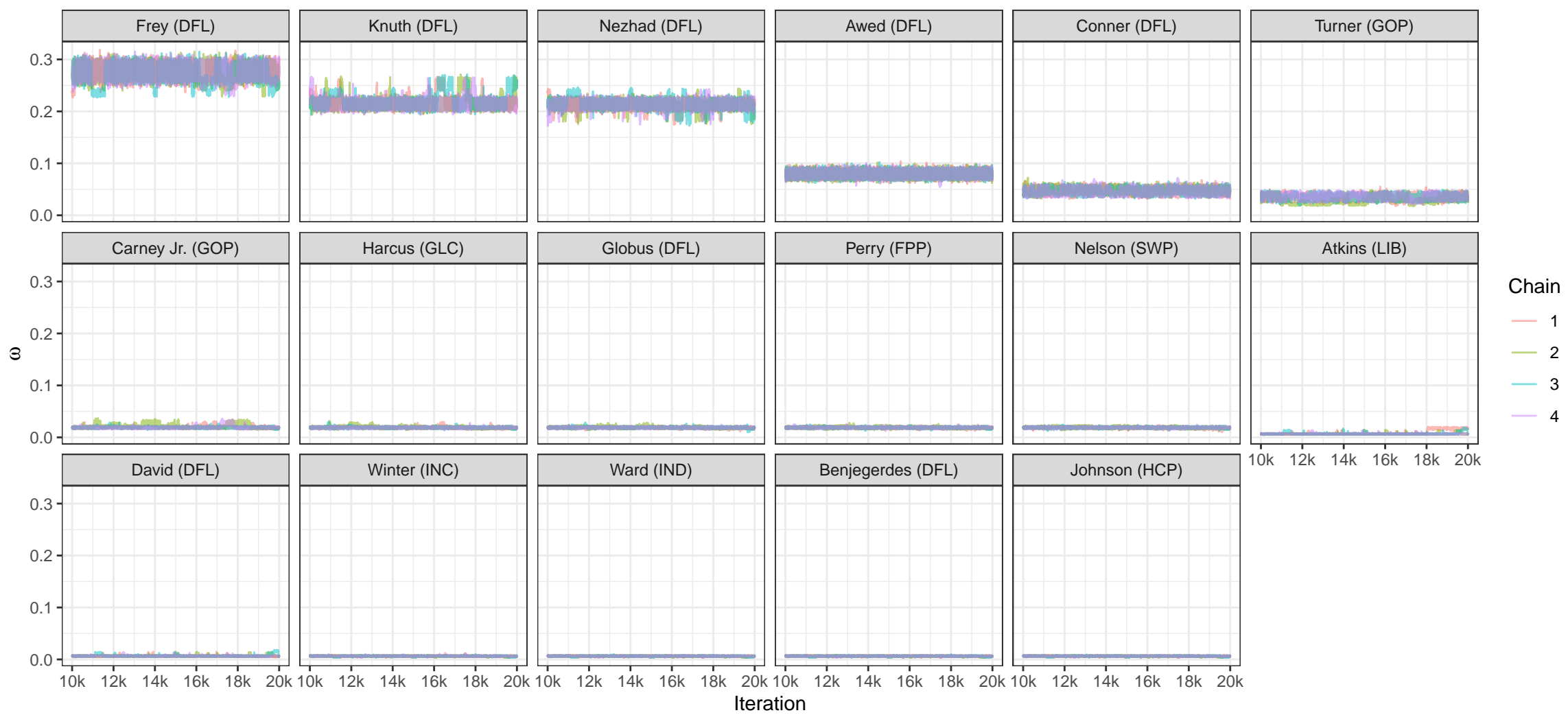

Supplement: Pearce and Erosheva supplementary material [file S0033312325100148sup001.zip › Figures/mayor_traceOmega.pdf]

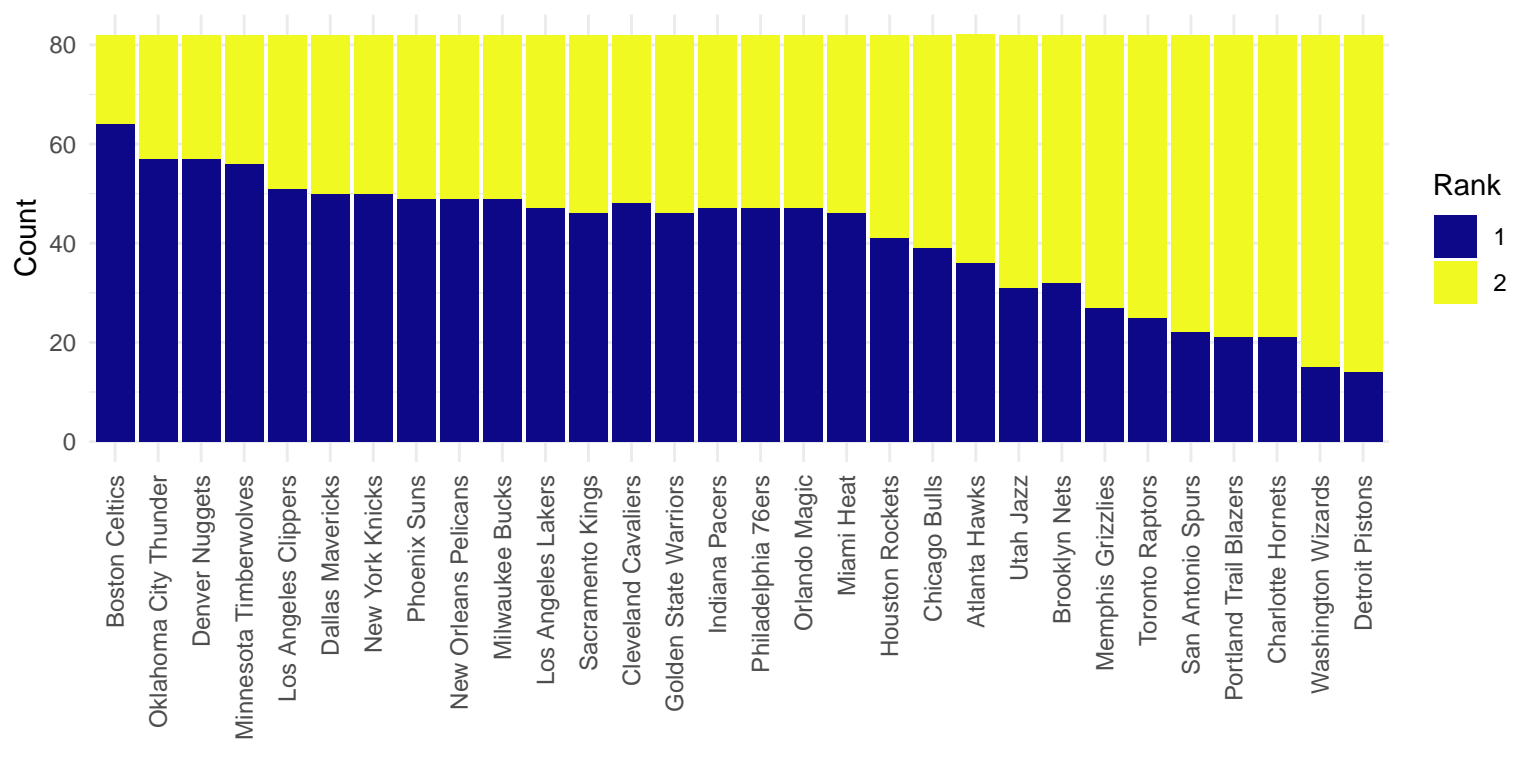

Supplement: Pearce and Erosheva supplementary material [file S0033312325100148sup001.zip › Figures/NBA_EDA.pdf]

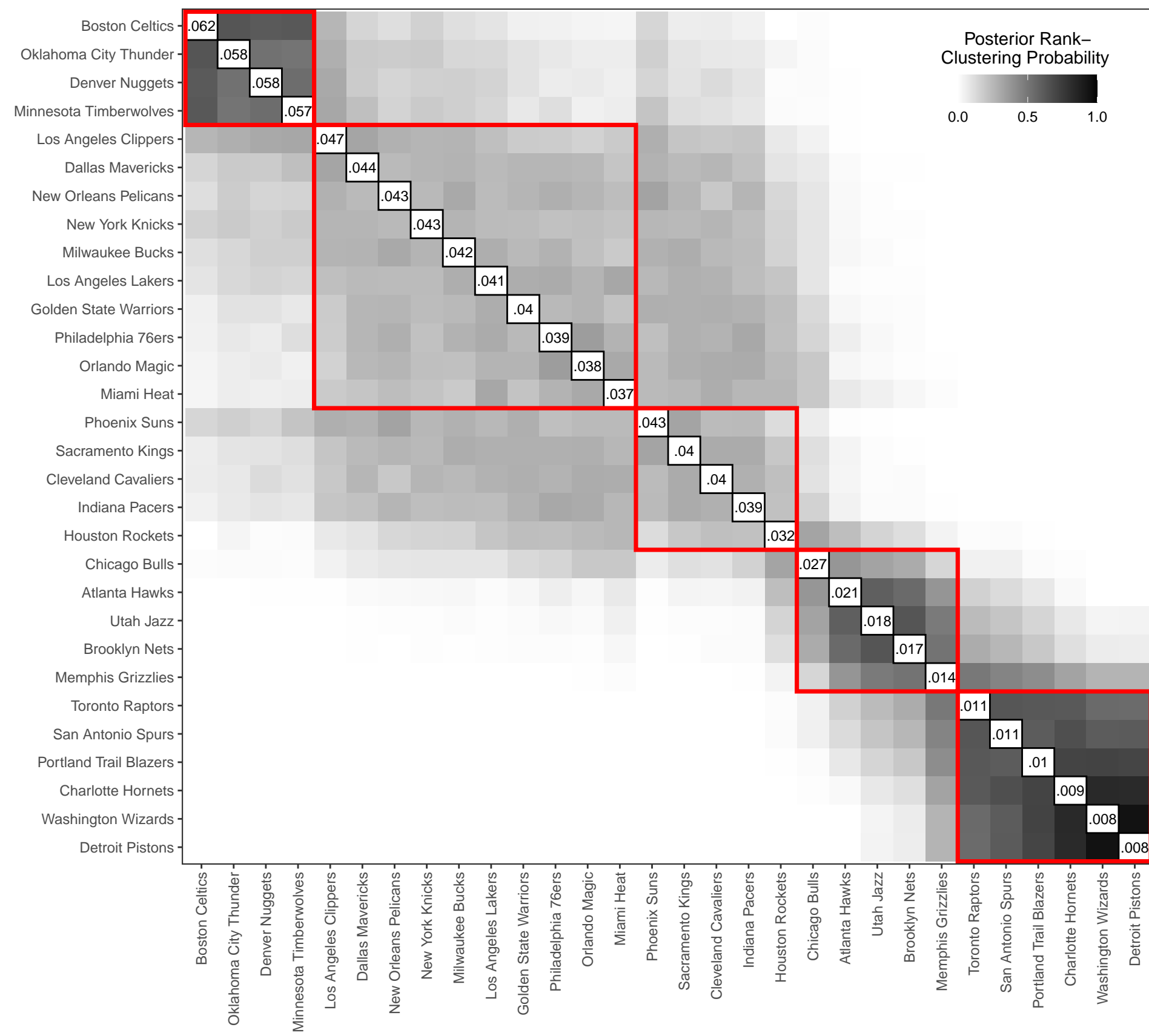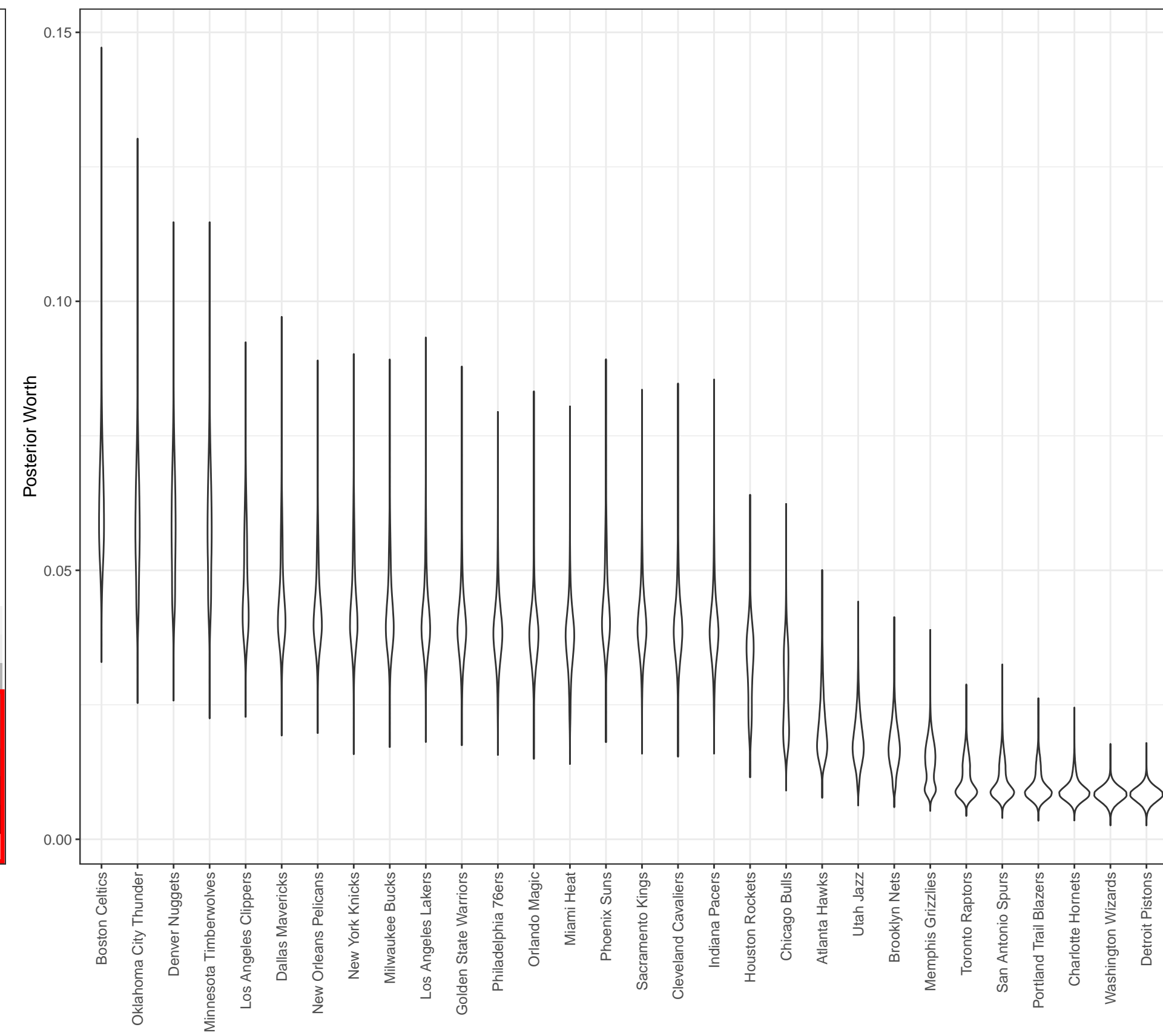

Supplement: Pearce and Erosheva supplementary material [file S0033312325100148sup001.zip › Figures/NBA_main.pdf]

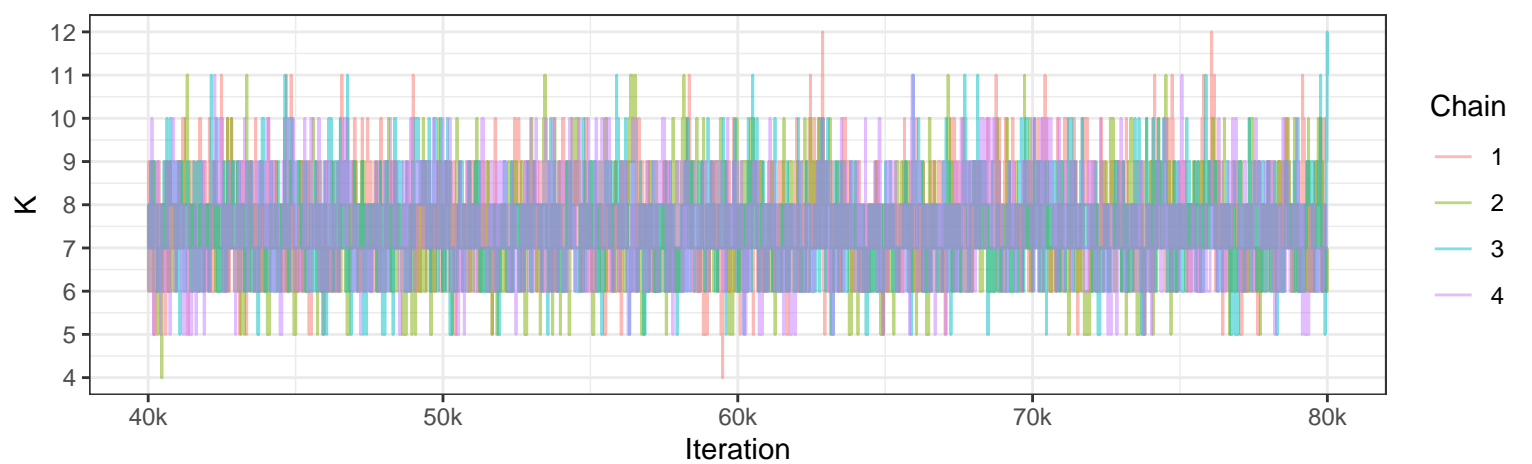

Supplement: Pearce and Erosheva supplementary material [file S0033312325100148sup001.zip › Figures/NBA_traceK.pdf]

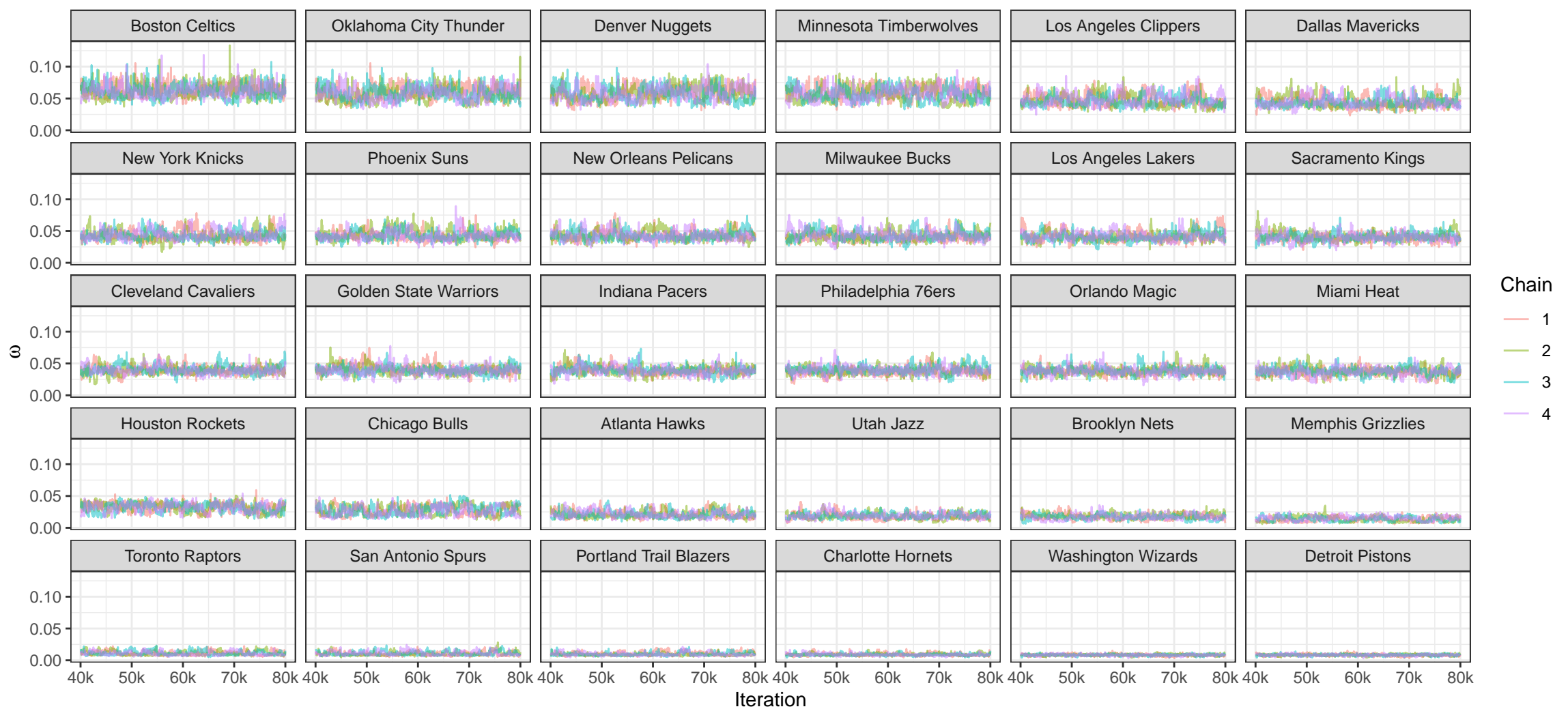

Supplement: Pearce and Erosheva supplementary material [file S0033312325100148sup001.zip › Figures/NBA_traceOmega.pdf]

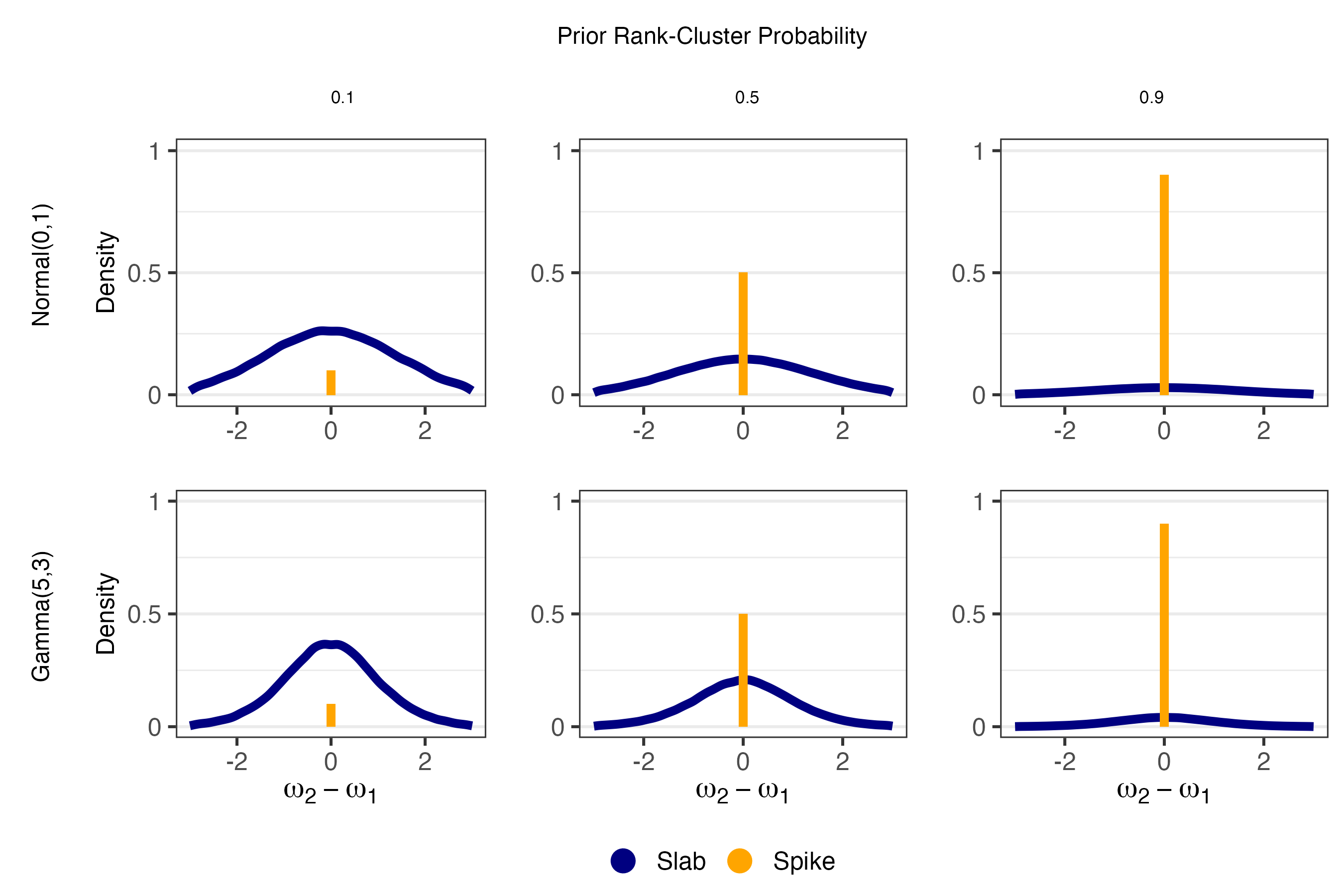

Supplement: Pearce and Erosheva supplementary material [file S0033312325100148sup001.zip › Figures/pdiff.png]

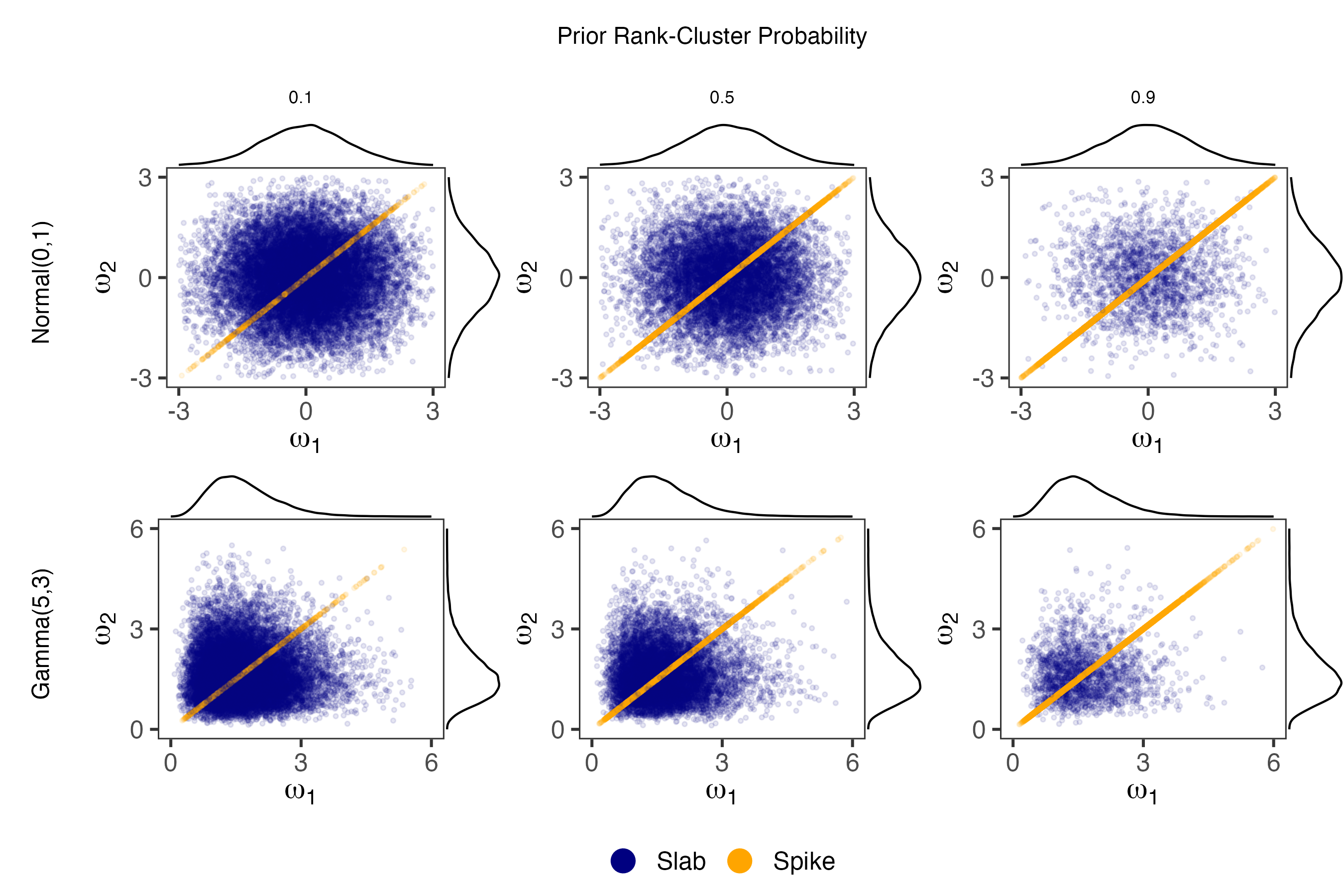

Supplement: Pearce and Erosheva supplementary material [file S0033312325100148sup001.zip › Figures/pssf_prior.png]

Posterior Mean Absolute Error

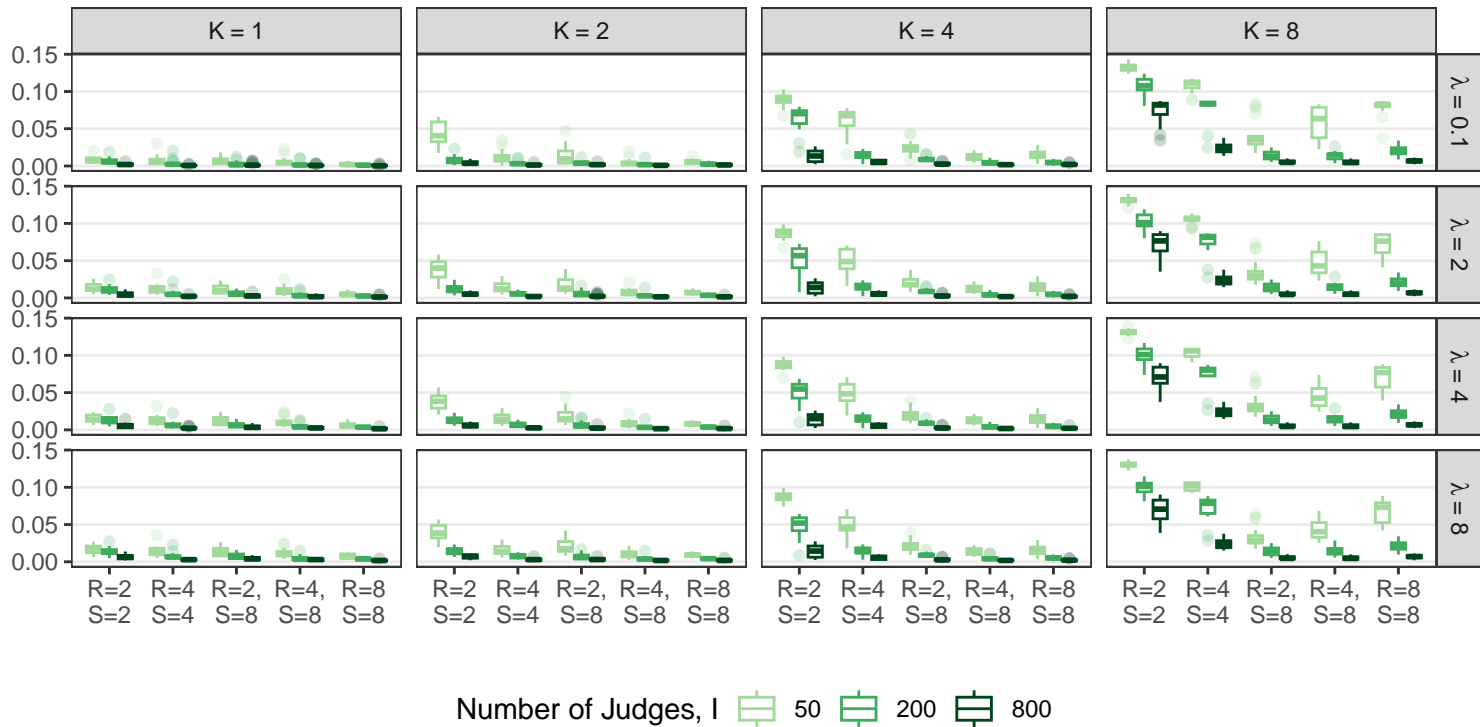

Supplement: Pearce and Erosheva supplementary material [file S0033312325100148sup001.zip › Figures/sim_MAE.pdf]

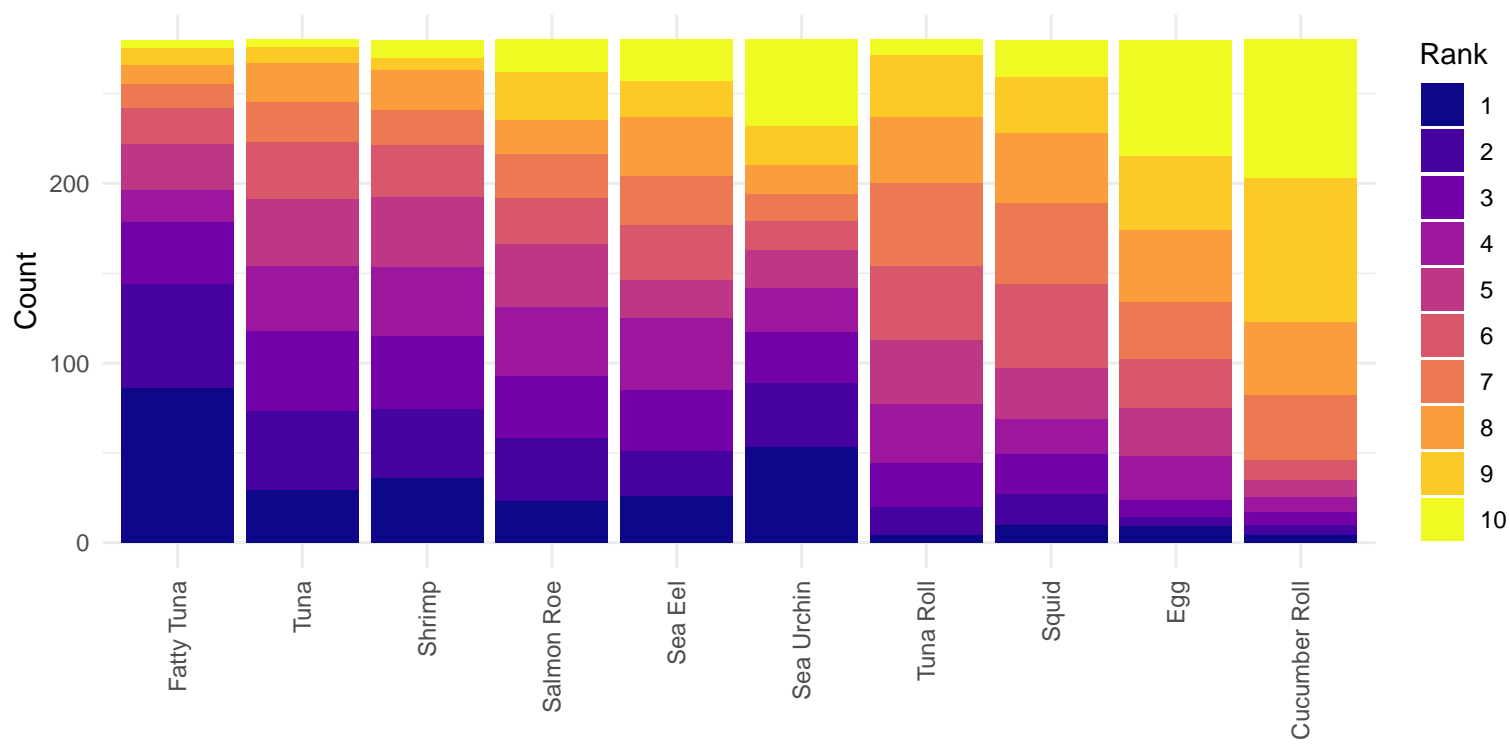

Supplement: Pearce and Erosheva supplementary material [file S0033312325100148sup001.zip › Figures/sushi_EDA.pdf]

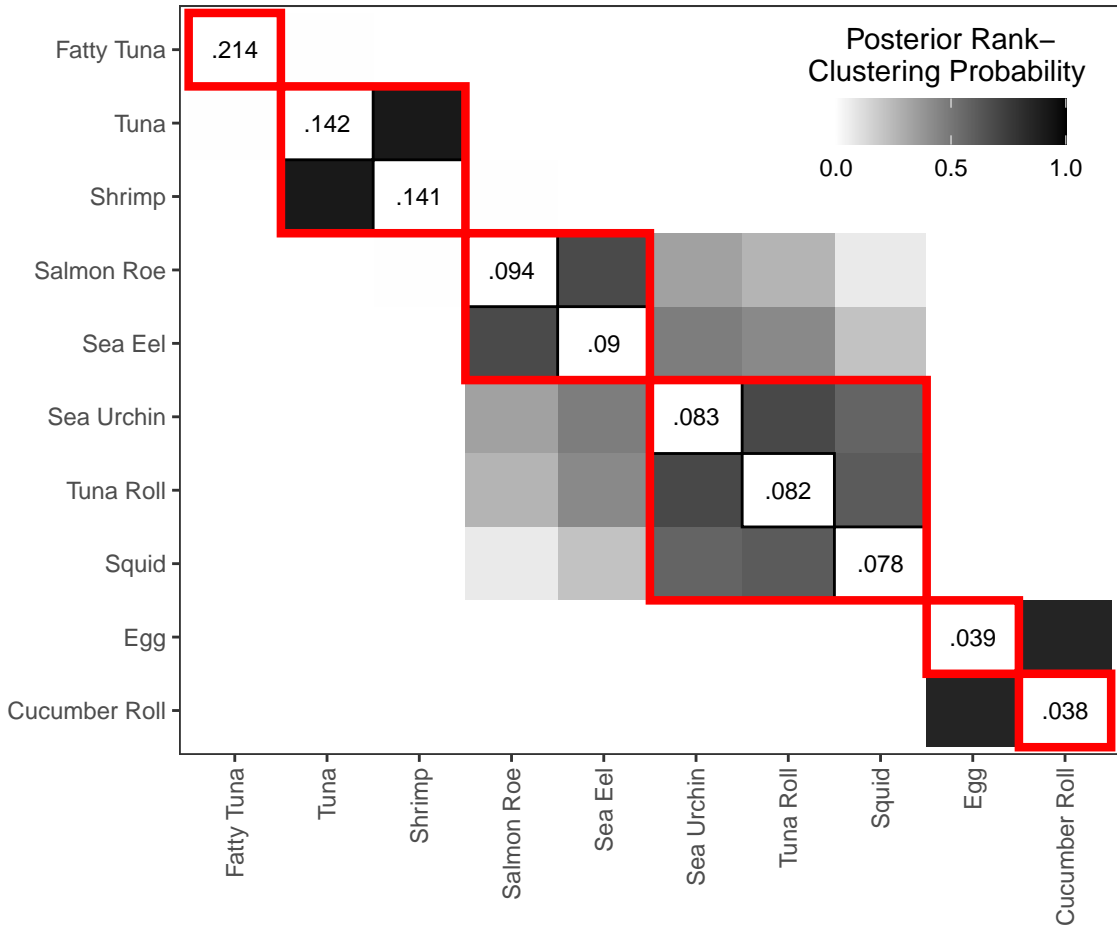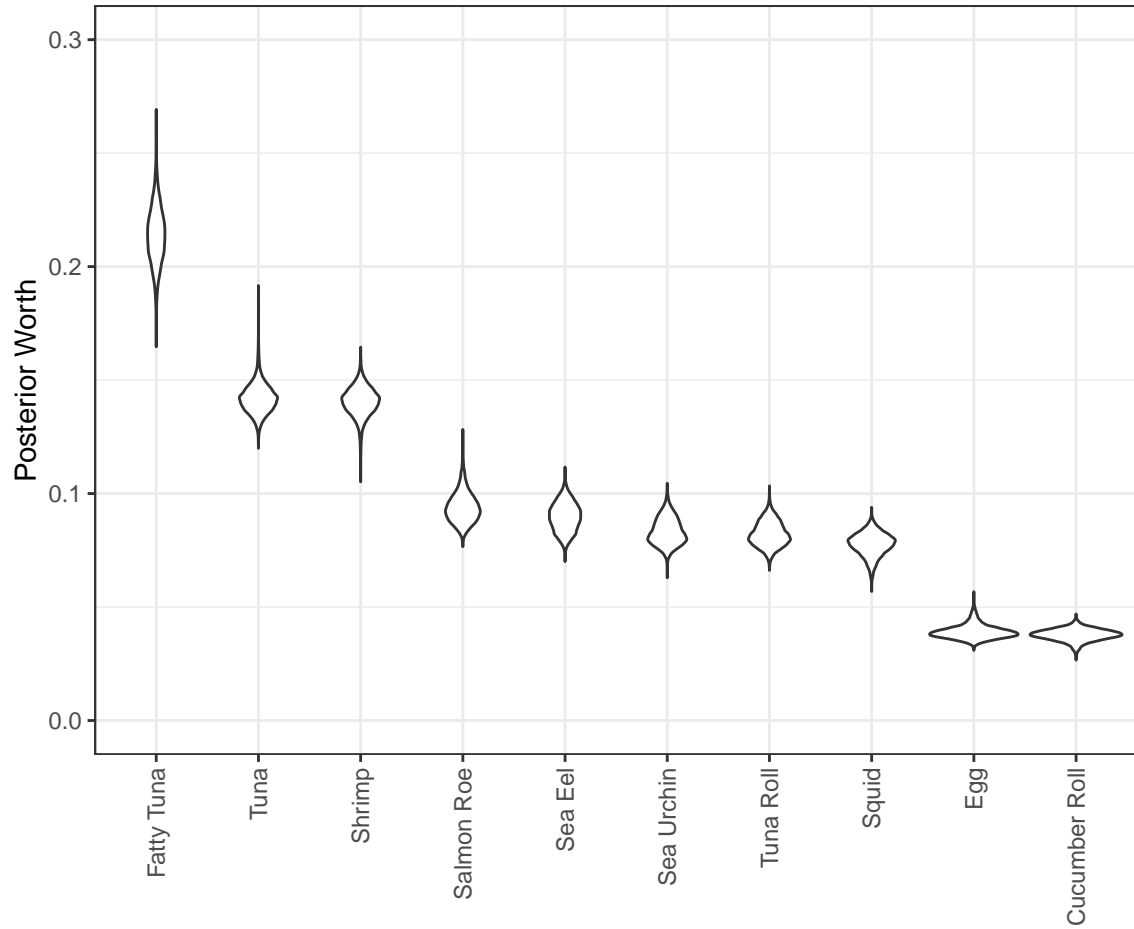

Supplement: Pearce and Erosheva supplementary material [file S0033312325100148sup001.zip › Figures/sushi_main.pdf]

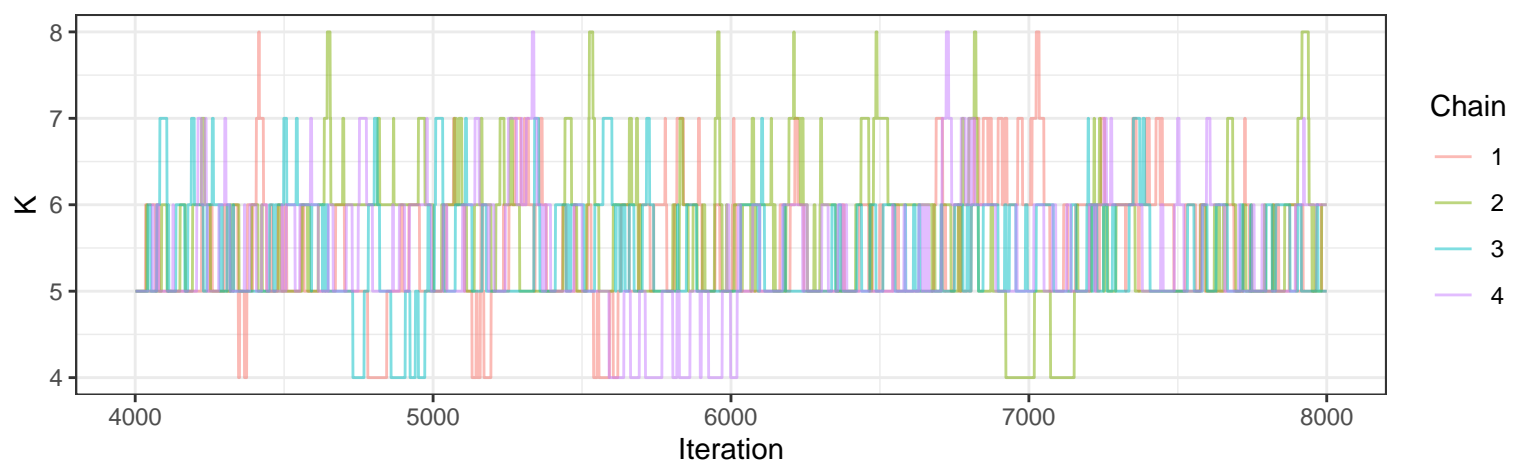

Supplement: Pearce and Erosheva supplementary material [file S0033312325100148sup001.zip › Figures/sushi_traceK.pdf]

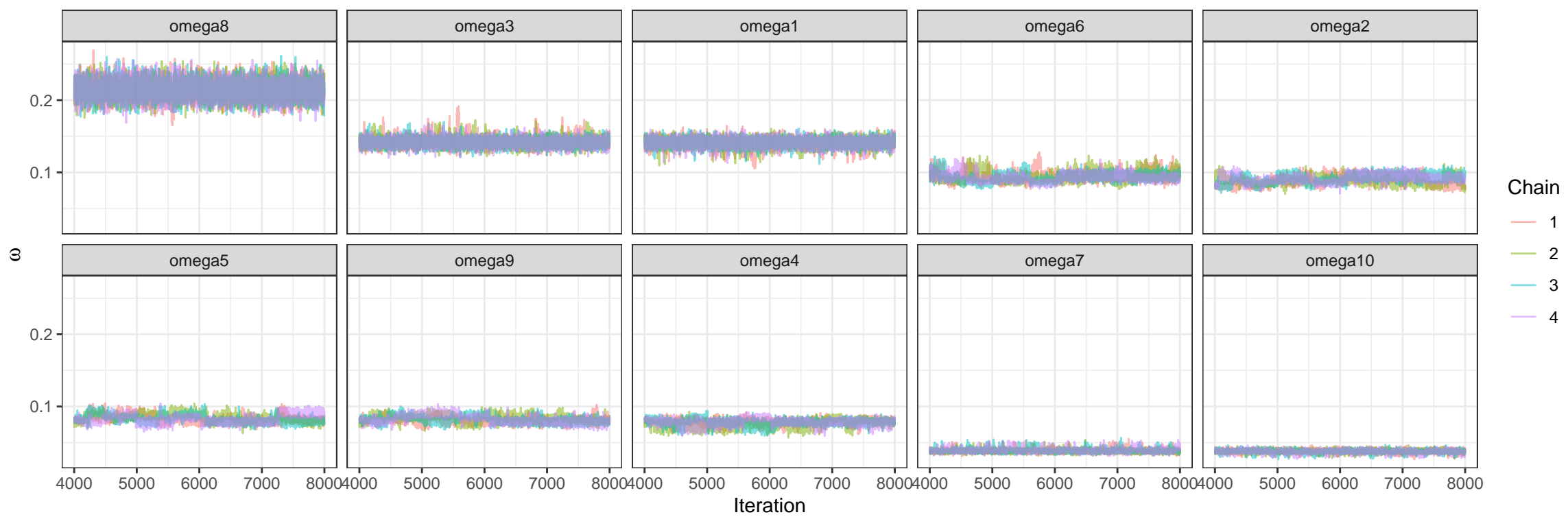

Supplement: Pearce and Erosheva supplementary material [file S0033312325100148sup001.zip › Figures/sushi_traceOmega.pdf]
